# Reference-guided *de novo* assembly approach improves genome reconstruction for related species

**DOI:** 10.1186/s12859-017-1911-6

**Published:** 2017-11-10

**Authors:** Heidi E. L. Lischer, Kentaro K. Shimizu

**Affiliations:** 1 Department of Evolutionary Biology and Environmental Studies (IEU), University of Zurich, Zurich, Switzerland; 2 Swiss Institute of Bioinformatics (SIB), Lausanne, Switzerland; 3 Kihara Institute for Biological Research, Yokohama City University, Yokohama, 244-0813 Japan

**Keywords:** Genome assembly, Reference-guided, De novo, Related species, Assembly evaluation

## Abstract

**Background:**

The development of next-generation sequencing has made it possible to sequence whole genomes at a relatively low cost. However, de novo genome assemblies remain challenging due to short read length, missing data, repetitive regions, polymorphisms and sequencing errors. As more and more genomes are sequenced, reference-guided assembly approaches can be used to assist the assembly process. However, previous methods mostly focused on the assembly of other genotypes within the same species. We adapted and extended a reference-guided de novo assembly approach, which enables the usage of a related reference sequence to guide the genome assembly. In order to compare and evaluate de novo and our reference-guided de novo assembly approaches, we used a simulated data set of a repetitive and heterozygotic plant genome.

**Results:**

The extended reference-guided de novo assembly approach almost always outperforms the corresponding de novo assembly program even when a reference of a different species is used. Similar improvements can be observed in high and low coverage situations. In addition, we show that a single evaluation metric, like the widely used N50 length, is not enough to properly rate assemblies as it not always points to the best assembly evaluated with other criteria. Therefore, we used the summed z-scores of 36 different statistics to evaluate the assemblies.

**Conclusions:**

The combination of reference mapping and de novo assembly provides a powerful tool to improve genome reconstruction by integrating information of a related genome. Our extension of the reference-guided de novo assembly approach enables the application of this strategy not only within but also between related species. Finally, the evaluation of genome assemblies is often not straight forward, as the truth is not known. Thus one should always use a combination of evaluation metrics, which not only try to assess the continuity but also the accuracy of an assembly.

**Electronic supplementary material:**

The online version of this article (10.1186/s12859-017-1911-6) contains supplementary material, which is available to authorized users.

## Background

In the last decade, the development of next-generation sequencing made it possible to obtain genome wide data at a relative low cost and in a short amount of time. This revolutionized the fields of genomics, transcriptomics, evolutionary biology and medical research. It is nowadays possible to sequence whole genomes of almost any organism at a decent coverage [1]. Reliable whole genome sequences are important for functional genomic analyses, genome wide scans for selections, assessing impact of genetic variations and rearrangements on evolution, study responses to environmental changes or gene expression [2]. It further provides the basis of genome wide linkage disequilibrium analyses, which are used to study population histories, identify signatures of selection in natural populations or the timing of admixture events [2–5].

Despite the decreasing cost of sequencing, it is still difficult and time consuming to de novo assemble reads into high-quality genomes [6, 7]. There exist powerful de novo assembly computer algorithms, which try to join reads into larger continuous contigs and use linkage information from mate-pair reads to extend them into even larger scaffolds. However, the generated reads are mostly short, contain errors and are unevenly distributed across the genome. Further, genomes may contain lots of repetitive regions, which are difficult to assemble and often cause errors leading to a lower quality of subsequent polymorphism analysis [7–9]. Diploid or polyploid organisms often contain a high degree of heterozygosity causing problems in the assembly process [10, 11], where heterozygous regions are frequently split into multiple contigs [12]. Thus, genome assemblies may result in incomplete and fragmented contigs/scaffolds containing misassembled regions and errors [2]. Recent studies start to use longer reads (e.g. using single-molecule real-time sequencing by Pacific BioSciences and single-molecule optical mapping by Bionano) to resolve repetitive regions and to create longer scaffolds [7, 13–16]. However, more difficult DNA extraction, high amounts of errors, and higher costs harbor additional problems and still limit their usage [1, 7, 17].

As more and more species get sequenced, there is the chance that the genome of a different but related species is already available, in which a significant proportion of the reads can be mapped. The genome of such a species, which we call closely related species, can then be used to assist the assembly of the target species. These so called reference-guided approaches make use of the similarity between target and reference species to gain additional information, which often lead to a more complete and improved genome [18–20]. Additionally, even genomes sequenced at a low coverage may provide useful genomic resources if they are guided by a reference genome [21, 22]. There are two main reference-guided assembly strategies: In the first one, reads are mapped against the reference genome and then used to construct an alternative consensus sequence [21]. This approach can be extended to polyploid genomes by using both diploid parents as references [11]. In the second approach, the reads are first de novo assembled. Afterwards, the resulting contigs/scaffolds are aligned against the reference genome to order and orientate them along chromosomes, to get gene information for genome annotation and to identify potential misassembled contigs or scaffolds [20, 21]. Sometimes, also a combination of the two approaches is applied [23]. However, the reference-guided assembly strategies have some disadvantages, as the resulting assemblies may contain some biases towards the used reference. More diverged regions may not be reconstructed and missing, and thus lead to a reduced diversity in the target assembly [13, 19, 21]. Additionally, errors in the reference sequence and chromosomal rearrangements between species may lead to mistakes [2]. All of these problems will accumulate with increasing divergence between reference and target species [22]. One solution to reduce these reference biases is to include multiple references of different strains or species [24, 25].

Schneeberger et al. [19] introduced an alternative reference-guided genome assembly approach to minimize the problems of reference biases. The main idea is to reduce the complexity of de novo assemblies with the aid of a reference sequence: First, homologous regions between target and reference genome are identified by mapping reads against the reference genome. These homologous regions are then used to define overlapping superblocks. Next, the reads are partitioned according these superblocks and separately de novo assembled. Additionally, also all unmapped reads are de novo assembled. In a further step, the reference genome is used to guide a Sanger assembler to merge the assembled contigs into nonredundant supercontigs. In a final step, supercontigs are error corrected with the original reads and scaffolded. This pipeline was developed for within species genome assemblies and therefore harbor some limitations in the usage of a reference genome from a different species. We adapted and modified the assembly approach and integrated an additional de novo assembly step after the redundancy removal to rescue divergent regions from getting lost. These modifications enable the use of a related genome to guide the assembly.

In this study, we investigate if our extended reference-guided de novo assembly approach using a related genome from a different species is able to outperform corresponding de novo assembly programs. In order to evaluate the assembly strategies, we simulated short Illumina reads from a repetitive and heterozygous genome. We also compare the results of de novo and reference-guided de novo assemblies in a low coverage situation. With the aim to get a final ranking between the genome assembly strategies, we applied a wide range of evaluation statistics accounting not only for continuity and completeness of the assembled genomes, but also for the number of errors and misassemblies.

## Methods

**Fig. 1:**
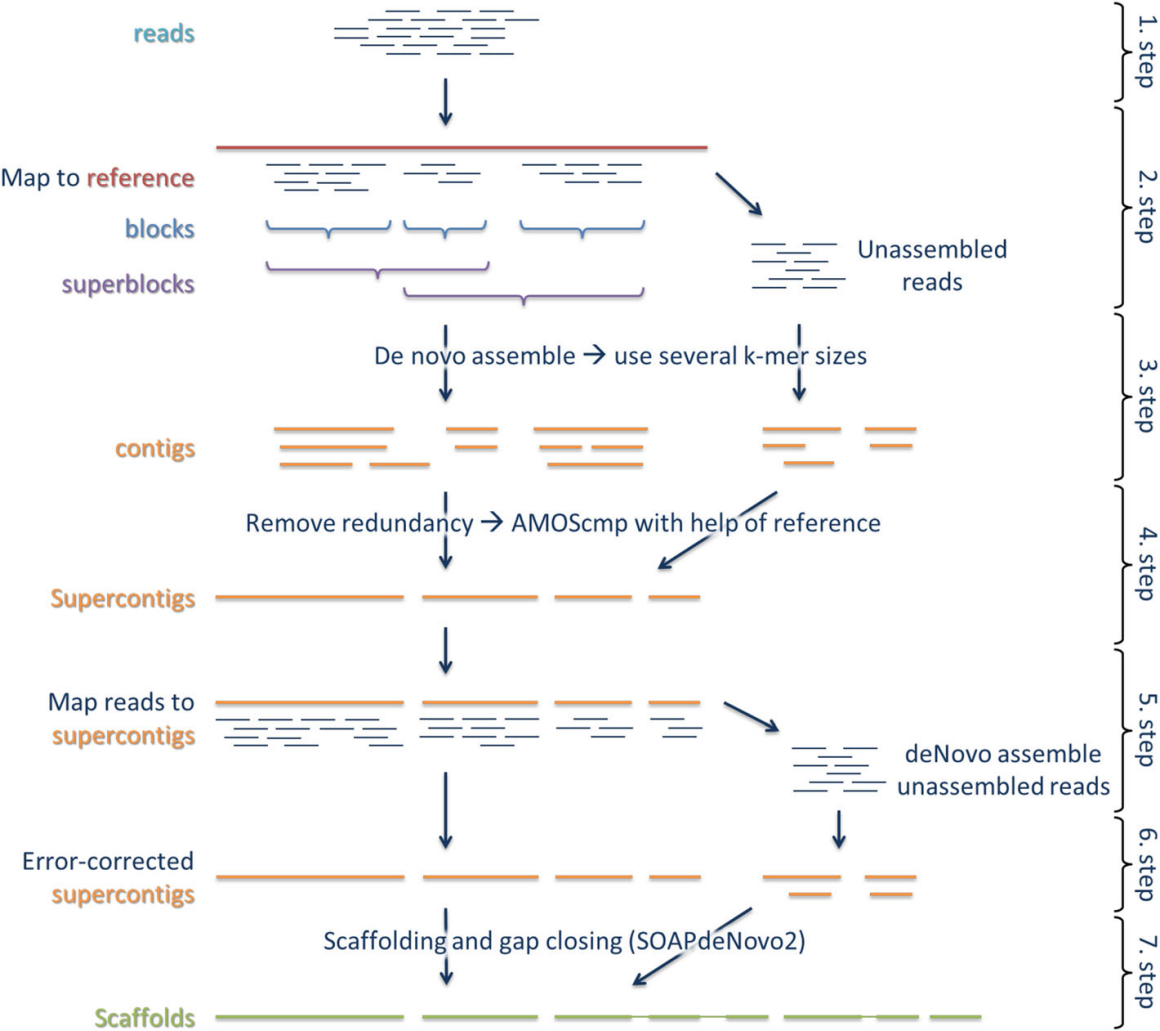
Reference-guided de novo assembly pipeline. Raw reads get quality trimmed (1. step) and mapped against a reference (2. step). Reference mapped reads are grouped into blocks with continuous read coverage. These blocks are then combined into superblocks until a total length of at least 12 kb is reached. Superblocks are overlapping by at least one block. Each superblock and all unmapped reads are separately de novo assembled (3. step). Resulting contigs are merged into non-redundant supercontigs (4. step). In the fifth step, reads are mapped back to the supercontigs and unmapped reads are de novo assembled to get additional supercontigs. All supercontigs are error corrected with back mapped reads (6. step) and afterwards used for scaffolding and gap closing (7. step)

We adapted and extended the reference-guided assembly approach from Schneeberger et al. [19]. The main idea of this approach is to first map reads against a reference genome of a related species to reduce the complexity of de novo assembly within continuous covered regions. In a further step, reads with no similarity to the related genome are integrated. In the next section we give a general overview of our reference-guided de novo assembly approach (for an illustration see Fig. 1), which can be used in combination with any de novo assembler.

### Reference-guided de novo assembly pipeline

In the 1th step, paired-end and optional mate-pair reads (mandatory if one plan to use an assembler which requires mate-pair reads, like ALLPATHS-LG [26]) are quality trimmed, and sequencing adapters and PCR primers are removed using Trimmomatic v0.32 [27]. Bases at the start and the end of a read are trimmed if they fall below a phred scaled quality threshold of 3. Additionally, reads are clipped if the average quality within a 4 bp sliding window falls below 15. Reads shorter than 40 bp are discarded. A final quality check is done using FastQC v0.10.1 [28]. In the second step, paired-end and mate-pair reads are mapped against an available reference genome of a related species using the fast-local mode of Bowtie2 v2.2.1 [29]. Afterwards, reads are assigned into blocks according to the previous alignment. A block is defined as a region with continuous read coverage. Blocks are extended if regions are spanned with at least 10 proper paired read pairs. Next, superblocks are defined based on the non-overlapping blocks. A superblock consists of the combination of two or more blocks until a total length of at least 12 kb is reached. Superblocks are overlapping by at least 300 bp by sharing one or more blocks with its neighbor superblock. If a superblock exceeds the maximal length of 100 kb, it is split into several superblocks of a maximal length of 100 kb and an overlap of 300 bp. The reason for this is to keep the later de novo assemblies within superblocks as simple and fast as possible. We identify the reads mapped to each superblock region and all unmapped reads with a mate mapped to the same region using samtools v1.3 [30]. In the third step, each superblock is separately de novo assembled with a de novo assembler of one’s own choice. If the de novo assembler requires the specification of a fixed k-mer, the de novo assembly of superblocks is repeated with different k-mer length. Additionally, all unmapped reads are de novo assembled to integrate highly diverged regions.

The resulting contigs contain some redundancy due to the overlapping nature of superblocks (and the repetition of de novo assemblies using different k-mer length). This redundancy is removed in the fourth step by assembling the contigs with the homology guided Sanger assembler AMOScmp v3.1.0 [18] using the same reference genome as in the second step. The AMOScmp scripts are run with default parameters except for casm-layout, in which we set the maximum ignorable trim length -t to 1000 and make-consensus where we use a minimum overlap -o of 10 bases. The resulting consensus sequences correspond to non-redundant supercontigs. Unfortunately, AMOScmp does not return any unassembled contigs and thus the most diverged contigs are lost. In order to get this information back, we align the trimmed reads back to the supercontigs using the sensitive mode of Bowtie2 (5. step). Next, all unmapped reads are de novo assembled and the resulting contigs are added to the list of supercontigs.

In order to validate and error correct the supercontigs, we align the trimmed paired-end reads against the supercontigs using the sensitive mode of Bowtie2 (6. step). Reads with a mapping quality lower than 10 are removed from the alignment. Additionally, a local realignment of reads around indels is done using GATK v3.1 [31] and Picard v1.109 [32]. Differences between reads and supercontigs indicate misassemblies and are corrected using samtools and bcftools v0.1.19 [30]. Furthermore, uncovered parts of superconitgs are removed and supercontigs are split using BEDTools2 v2.19.1 [33] and an in house program. Any supercontig shorter than 200 bp is discarded. In the final step, trimmed paired-end and mate-pair reads are used in the ranked scaffolding and gap closing using SOAPdenovo2 vr240 [34]. Scaffolds shorter than 1 kb are discarded.

### Application of the reference-guided de novo assembly pipeline on a simulated data set

In order to evaluate the reference-guided de novo assembly approach we needed two genomes of related organisms. The first one was used to simulate reads and to evaluate resulting genome assemblies. The second genome was needed to guide the assembly in the reference-guided de novo assembly approaches. For this purpose, two species with chromosome-scale genome assemblies, that are closely related but with considerable rearrangements would be most suitable. Therefore, we chose the *Arabidopsis lyrata* [35, 36] and the *Arabidopsis thaliana* (TAIR10) genomes [37, 38]. Phylogenomic studies showed that *Arabidopsis thaliana* (2*n* = 10) is clearly separated from *A. lyrata* (*2n* = 16) at the gene tree level [39] and they diverged between ~5–22.7 million years ago [40, 41]. Their genomes not only differ largely in size (*A. thaliana* as a typical predominantly selfing species has a reduced size of 125 Mb, compared to *A. lyrata* with a genome size of 205 Mb), but also in many rearrangements [35]. Transposable elements largely contribute to the reduced genome size of *A. thaliana* [42, 43]. More than 50% of the *A. lyrata* genome is missing in the *A. thaliana* genome and the sequence similarity is only around 80% in common regions [35].

We used the next-generation sequencing read simulator ART version VanillaIceCream-03-11-2014 [44] to simulate 100 bp long paired-end Illumina reads of the *A. lyrata* genome with an insertion size of 150, 200 and 400 bp (standard deviation of 34, 36 and 87 bp) and a 72, 72 and 40 fold coverage. Furthermore, ART was used to simulate 100 bp long mate-pair Illumina reads with a 76, 82, 104, 44 and 40 fold coverage and an insertion size of 3, 5, 7, 11 and 15 kb with a standard deviation of 400 bp. In order to simulate heterozygosity, half of the paired-end and mate-pair reads of each library were simulated from a modified *A. lyrata* genome, where we randomly exchanged 1% of any non-N bases by any other of the 3 bases.

The simulated reads were used to assemble the *A. lyrata* genome applying the reference-guided de novo assembly pipeline using *A. thaliana* genome as a reference. We tested the pipeline with four different de novo assemblers: SOAPdenovo2 vr240 [34], ABySS v1.3.7 [45], IDBA-UD v.1.1.1 [46] and ALLPATHS-LG [26]. In the pipelines using ABySS and SOAPdenovo2, step 3 (the de novo assembly of superblock and unmapped reads) was repeated five times using five different k-mers sizes: 41, 51, 61, 71 and 81 bp. Additionally, the de novo assembly in step 5 was done using a k-mer size of 61 bp. The reference-guided de novo assembly pipelines of the four assemblers can be downloaded from https://bitbucket.org/HeidiLischer/refguideddenovoassembly_pipelines. In order to test the influence of a closer related genome, we additionally run the reference-guided de novo assembly pipeline with ALLPATHS-LG using the original *A. lyrata* genome as reference.

Furthermore, we run the pipeline under a low coverage situation using either ABySS, SOAPdenvo2, ALLPATHS-LG or IDBA-UD assembler and *A. thaliana* as a reference. For this reason, 10% of each simulated paired-end and mate-pair library were subsampled using the Seqtk v1.0-r45 [47]. The de novo assembly step 5 of ABySS and SOAPdenvo2 was run using a k-mer size of 51 bp. The main modification we introduced into the reference-guided approach of Schneeberger et al. [16] is the additional de novo assembly step after the redundancy removal (Fig. 1, step 5). In order to check the influence of this modification, we additionally run the pipeline without this step 5 using the low coverage simulated data set and either of the four assemblers.

### De novo assembly of a simulated data set

In order to compare reference-guided de novo assembly approaches with classical de novo assemblies, we used the same simulated paired-end and mate-pair reads from the *A. lyrata* genome to run de novo assemblies using the same softwares: SOAPdenovo2, ABySS, IDBA-UD and ALLPATHS-LG. All simulated reads were first quality trimmed and adapters removed like in step 1 of the reference-guided de novo assembly pipeline. ABySS and SOAPdenovo2 were run with a k-mer size of 71 bp and within SOAPdenovo2 a ranked scaffolding and gap closing was done. Note that mate-pair libraries were only used in the scaffolding process except for ALLPATHS-LG. Resulting scaffolds shorter than 1 kb were discarded as in the reference-guided de novo assembly approach.

Additionally, we also tested the de novo assembly performances of ABySS, SOAPdenvo2, IDBA-UD and ALLPATHS-LG with the low coverage simulated data set, in which ABySS and SOAPdenovo2 were run with a k-mer size of 51 bp.

### Evaluation of de novo and reference-guided de novo assemblies

We used several statistics and tools to compare and evaluate all de novo and reference-guided de novo assemblies using the original *A. lyrata* genome sequence as the correct reference. First we reported the number and N50 (length of the contig that using equal or longer contigs sum up to half of the assembly length) of all contigs. Additionally, we measured the absolute difference between the length of the *A. lyrata* genome and the total length of all gene-sized contigs (> = 1.2 kb), analog to Bradnam et al. [7]. We used the Ensembl Plant Mart *A. lyrata* genes (v. 1.0) dataset [48] to calculate the size of an average *A. lyrata* gene. We also estimated the NG50 (length of the contigs that using equal or longer contigs sum up to half of the *A. lyrata* genome length [49]) using the genome assembly gold-standard evaluations tool GAGE [6]. Additionally, the number of misassemblies (translocations: number of sequences in a contig/scaffold which map on different reference chromosomes; relocations: number of sequences in a contig/scaffold which map >1 kb apart from each other or overlap by >1 kb; inversions: number of sequences in contig/scaffold which map on opposite strands of the same chromosome), duplication ratio and the number of covered genes was estimated using the quality assessment tool QUAST with the *A. lyrata* genome as a reference [50].

In a next step, we evaluated the scaffolds by reporting number and N50 of all scaffolds. We also estimated the absolute length differences between the *A. lyrata* genome and the total length of all scaffolds, as well as between the genome and the total length of gene-sized scaffolds (> = 1.2 kb). We mapped the trimmed paired-end reads back to the scaffolds using the sensitive mode of Bowtie2 and calculated the percentage of mapped reads, mapped reads with a mapping quality > = 10 and the percentage of proper paired reads with a mapping quality > = 10 using samtools v0.1.19 and bamTools v2.3.0 [51]. We calculated the scaffold NG50 and the error corrected NG50 using GAGE. The error corrected NG50 corresponds to the NG50 value computed on sequences broken at each misassembly. Additionally, we estimated the relative length of the error corrected NG50 and NG50. We also analyzed the scaffolds using QUAST to estimate the average number of N’s per 100kbp, number of misassemblies (translocations, relocations and inversions), percentage of misassembled scaffolds, the percentage of misassembled scaffold length, number of local misassemblies (two or more scaffolds map to the same position or the gap between left and right flanking sequence is less than 1 kb apart), the percentage of unaligned scaffolds, the duplication ratio, the average number of indels per 100 kb and the number of covered genes. We used CEGMA tool [52, 53] to assess the presence of the 458 core eukaryotic genes and the 248 most highly conserved and at least paralogous core eukaryotic genes. Additionally we run compass [7, 54] to estimate the genome coverage, validity (fraction of the assembly which can be validated by the reference), multiplicity and parsimony (cost of the assembly; assembled versus validated bp) of the scaffolds. We also applied two evaluation tools which are independent of any reference sequence, instead they use read alignments for assembly evaluations: the generic assembly likelihood framework ALE [55] and the universal genome assembly evaluation tool REAPR v1.0.18 [56]. ALE scores were estimated based on the alignments of the 200 and 400 bp insertion paired-end libraries against the scaffolds. We run REAPR smaltmap pipeline to map the 200 bp insertion paired-end library and 7 kb insertion mate-pair library against the scaffolds. The REAPR perfectfrombam was used to get perfect uniquely mapped reads from the 200 bp paired-end mapping using a 50 bp lower insertion and a 350 bp upper insertion bound, a maximum mapping quality of 3 to identify repetitive regions, a perfect minimum quality score of 4 and perfect minimum alignment score of 90. This was then used together with the 7 kb mate-pair mapping to run the REAPR pipeline to get the number of errors and estimate a REAPR score (fraction of error free bp * broken N50 length / N50).

In order to summarize the 36 different evaluation statistics and compare the different assemblies, we calculated z-scores for each statistic analog to Bradnam et al. [7]. The z-scores correspond to how many standard deviations a value is away from the mean over all evaluated assembly methods. To rank the assembly methods, the z-scores of all statistics are summed. Error bars correspond to the best and worst summed z-score if one statistic was omitted. Violin plots from z-scores were generated using the vioplot function of the vioplot package of R [57, 58]. A one sided Wilcoxon rank sum test over z-scores was used to test if a higher ranked assembly method was significant better than the other assembly method using the R wilcox.test function [57]. The evaluation of the low coverage assemblies was done using the same statistics.

## Results

**Fig. 2:**
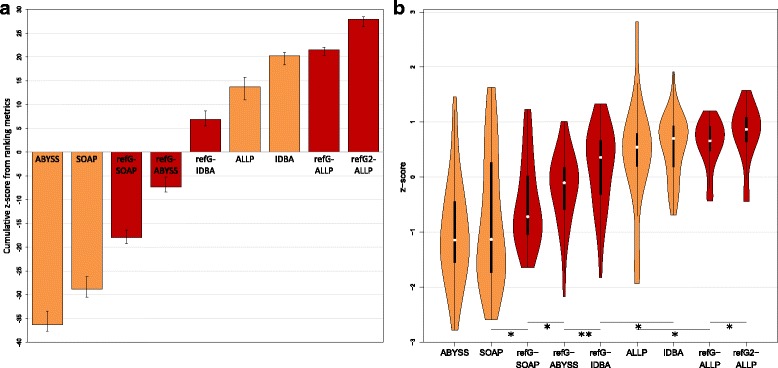
Z-score ranking based on 36 evaluation statistics. The cumulative z-score ranking (**a**) based on 36 evaluation statistics between different assembly approaches. Error bars correspond to the best and worst summed z-score that could be reached by omitting one evaluation statistic from the analysis. De novo assembly programs are shown in orange and reference-guided de novo assembly approaches in red (refG2 corresponds to the approach guided by the closer *A. lyrata* genome). The violin plots of z-scores are shown in (**b**) in which the white points correspond to medians, black boxes to interquartile ranges and the orange/red areas to the kernel density estimations of the z-scores. The lines and stars indicate significant higher z-scores (*: *p*-value <0.05, **: *p*-value <0.01)

In order to evaluate de novo and reference-guided de novo assembly strategies, we simulated 332,721,052 paired-end reads (130 million reads per 150 bp and 200 bp insertion library and 72 million reads with 400 bp insertion) and 616,924,410 mate-pair reads (3 kb insertion: 136; 5 kb: 146; 7 kb: 185; 11 kb: 78; 15 kb: 70 million reads) from the *A. lyrata* genome. We used 36 different evaluation statistics to assess the performance of the different assembly strategies (see Additional file 1). Fig. 2 gives an overview of the final ranking of the assembly approaches according to the summed z-scores over all evaluation statistics. Here we report the approaches from the worst to the best assemblies: Generally, the reference-guided de novo assembly approaches performed better than the corresponding de novo assemblies, except for the IDBA-UD assembler. The ABySS and SOAPdenovo2 de novo assemblers resulted in the worst assemblies, whereas SOAPdenovo2 was slightly but not significant better (*p*-value = 0.3572). Using the reference-guided de novo assembly approach with SOAPdenovo2 led to significant (*p*-value = 0.0336) better result than the SOAPdenovo2 de novo assembly. Further improved assemblies were reached by the reference-guided de novo assembly using ABySS (comparison with reference-guided SOAPdenovo2: *p*-value = 0.0228) and IDBA-UD (comparison with reference-guided ABySS: *p*-value = 0.0063). The de novo assembly of ALLPATHS-LG was slightly but not significantly (*p*-value = 0.1567) better than the reference-guided de novo assembly of IDBA-UD. The de novo IDBA-UD assembly was slightly (not significantly, *p*-value = 0.1026) better than the de novo ALLPATHS-LG assembly. However, the de novo IDBA-UD assembly was significant better than the reference-guided assembly with IDBA-UD (*p*-value = 0.0115). The second best assembly was the reference-guided de novo assembly using ALLPATHS-LG. It did not significantly (*p*-value = 0.4708) improve compared to the de novo IDBA_UD, but was significant better than the de novo ALLPATHS-LG (*p*-value = 0.0409). Overall the best performance in the assembly of the heterozygous reads showed the reference-guided de novo assembly of ALLPATHS-LG using the original haploid *A. lyrata* genome as a reference (*p*-value = 0.0181).

**Fig. 3:**
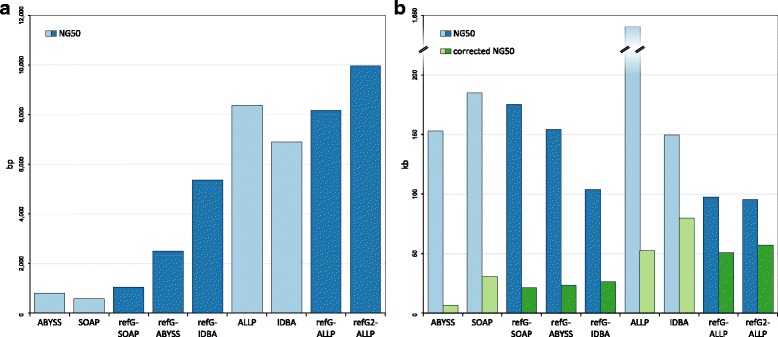
NG50 values of different assembly approaches. Contig NG50 (**a**) and scaffold NG50 (**b**) values of the different assembly approaches. De novo assembly programs are shown in light blue and reference-guided de novo assembly approaches in dark blue (refG2 corresponds to the approach guided by the closer *A. lyrata* genome). Additionally, (**b**) shows the corrected scaffold NG50 values in green (de novo: light green, reference-guided de novo assembly approaches: dark green)

**Fig. 4:**
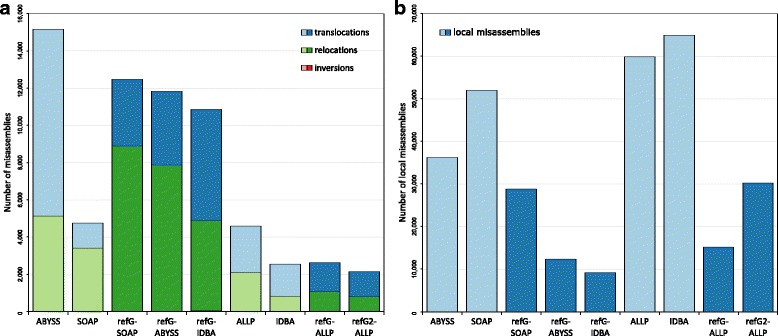
Number of misassemblies. Number of translocations (blue), relocations (green) and inversions (red) of the different assembly approaches are shown in (**a**). De novo assembly programs are shown in light colors and reference-guided de novo assembly approaches in dark colors (refG2 corresponds to the approach guided by the closer *A. lyrata* genome). Numbers of local misassemblies are shown in (**b**)

**Fig. 5:**
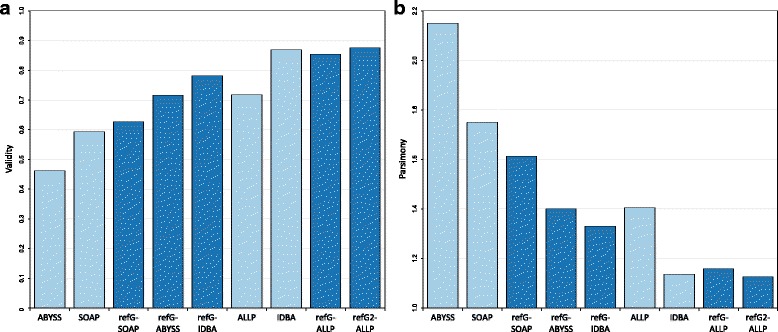
Validity and parsimony (cost) of different assembly approaches. Validity (**a**) and parsimony (**b**) of the different assembly approaches. De novo assembly programs are shown in light blue and reference-guided de novo assembly approaches in dark blue (refG2 corresponds to the approach guided by the closer *A. lyrata* genome). Validity correspond to the fraction of the assembly which can be validated by the reference and parsimony (cost) to the assembled versus validated bp

If we have a closer look at the different evaluation statistics the ranking within one metric can be very different. While the contig NG50 more or less showed the same order as the overall ranking (Fig. 3a), the scaffold NG50 had a very different ranking (Fig. 3b). Especially ALLPATHS-LG had an extremely large NG50 scaffold length of 1.6 Mb, which is more than 8 times larger than the second largest NG50 of the SOAPdenovo2 assembler (185 kb). However, the error corrected NG50 length of GAGE was in the range of the other assemblers, indicating that it encompass a large number of misjoined scaffolds. The number of misassemblies estimated by QUAST was lowest in the reference-guided de novo assembly using the *A. lyrata* as reference, followed by the IDBA-UD de novo assembly and the reference-guided de novo assembly with ALLPATHS-LG (Fig. 4a). Most of the misassemblies were due to translocations and relocations, whereas inversions were overall quite rare. Generally, the reference-guided de novo assembly approaches had fewer local misassemblies than the corresponding de novo assemblies (Fig. 4b). The evaluation with COMPASS revealed an *A. lyrata* genome coverage between 60 and 73% (Additional file 1), in which the reference-guided de novo assemblies had an overall higher coverage compared to the corresponding de novo assemblies. The validity and the cost (assembled bp versus the validated bp) of assemblies were highest and lowest, respectively, in the two reference-guided de novo assemblies using ALLPATHS-LG and the IDBA-UD de novo assembly (Fig. 5). Overall, the reference-guided de novo assemblies had a higher validity and a lower cost than the corresponding de novo assemblies, except for the de novo IDBA-UD assembler.

**Fig. 6:**
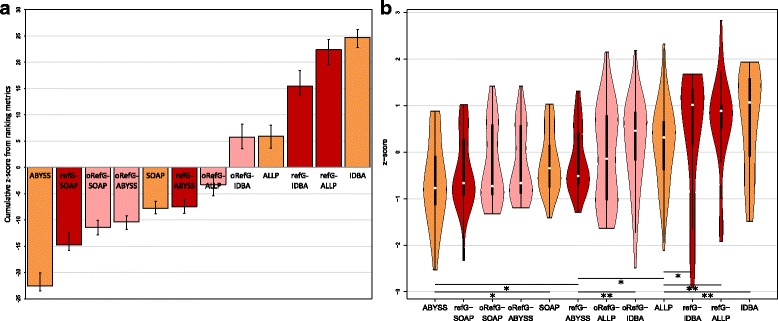
Low coverage z-score ranking based on 36 evaluation statistics for de novo and reference-guided de novo assembly approaches with and without step 5. The cumulative z-score ranking (**a**) based on 36 evaluation statistics between the different low coverage assembly approaches. Error bars correspond to the best and worst summed z-score that could be reached by omitting one evaluation statistic from the analysis. De novo assembly programs are shown in orange and reference-guided de novo assembly approaches with step 5 (refG) in red and without step 5 (oRefG) in light red. The violin plots of z-scores from the low coverage data set are shown in (**b**) in which the white points correspond to medians, black boxes to interquartile ranges and the orange/red areas to the kernel density estimations of the z-scores. The lines and stars indicate significant higher z-scores (*: *p*-value <0.05, **: *p*-value <0.01)

All the assembly approaches (except the reference-guided de novo assembly approach using the *A. lyrata* genome as a reference) were also tested with a low coverage data set using only 10% of all simulated reads. As expected, the assemblies were overall much poorer than the assemblies with the complete data set (see Additional files 1 and 2). Fig. 6 shows the overall ranking of the low coverage approaches. The ABySS and SOAPdenovo2 de novo assemblers and the reference-guided de novo assembly using SOAPdenovo2 resulted in the worst assemblies. Whereas SOAPdenovo2 was slightly but not significant better than the reference-guided de novo assembly using SOAPdenovo2 (*p*-value = 0.4347) and this approach again was slightly but not significant better than the ABySS de novo assembly (*p*-value = 0.1224). However, the SOAPdenovo2 de novo assembly was significant better than the de novo assembly of ABySS (p-value = 0.0117). In addition, the reference-guided de novo assembly using ABySS performed better than the ABySS de novo assembly (p-value = 0.0103). The ALLPATHS-LG de novo assembler led to a significant better assembly than the reference-guided de novo assembly using ABySS (*p*-value = 0.0270). A further improvement was reached using either the reference-guided de novo assembly approach with IDBA-UD (*p*-value = 0.0117) or ALLPATHS-LG (p-value = 0.0027) or the IDBA-UD de novo assembler (*p*-value = 0.0066). The IDBA-UD de novo assembler performed slightly but not significant better than the reference-guided de novo assembly using ALLPATHS-LG (*p*-value = 0.1924) or IDBA-UD (*p*-value = 0.1782).

Additionally, the low coverage data set was used to compare our reference-guided de novo assembly approach with and without (similar to the original approach) the de novo assembly step 5 (see Fig. 6 and Additional file 2). In the approach using either ABySS or SOAPdenovo2, the reference-guided de novo assemblies with and without step 5 were not significantly different from each other (ABySS: *p*-value = 0.0922; SOAPdenovo2: *p*-value = 0.4347). However, the overall assembled genome length and N50 was much larger if the approach was run with the additional de novo assembly step 5 (see Additional file 2). Using the overall better assemblers IDBA-UD and ALLPATHS-LG, the integration of the step 5 within the reference-guided de novo assembly pipeline led to significant improved assemblies (IDBA-UD: *p*-value = 0.0078; ALLPATHS-LG: *p*-value = 0.0038).

## Discussion

The evaluation with a simulated data set shows that our reference-guided de novo assembly approach leads in almost all cases to a better genome assembly than the corresponding de novo assembly (see Fig. 2 and Additional file 1). Similar improvements can also be observed in a low coverage situation (see Fig. 6 and Additional file 2). The overall best assembly can be achieved with our reference-guided de novo assembly pipeline using ALLPATHS-LG. However, one should be aware that this is not an ultimate ranking. Other studies have shown that assemblers may perform quite differently on varying data sets and species [7]. The performance of assembly programs and algorithms is strongly influenced by the level of coverage, heterozygosity, repetitions, errors, but also the library compositions (e.g.: insertion lengths) [7]. Therefore, an elaborated evaluation of each genome assembly is required and one should always run and compare different assembly programs and approaches.

The overall best de novo assembly was produced by the IDBA-UD assembler. It is also the only example where the de novo assembly outperformed the corresponding reference-guided de novo assembly approach (see Fig. 2 and Additional file 1). IDBA-UD was especially designed for the assembly of genomes with uneven coverage and it also outperformed other tools in metagenomics assemblies of a microbial communities [46]. Metagenomic assemblies have to deal with many differences between genomes, which can somehow be comparable to heterozygous sites in diploid/ployploid genomes. Thus, IDBA-UD seems not only to perform good in metagnomic assemblies, but also in genome assemblies with a large fraction of heterozygous positions like in our simulated data set with 1% heterozygosity. However, IDBA-UD requires a large amount of memory in the assembly process. Already the de novo assembly of the relatively small 200 Mb *A. lyrata* genome required 355 GB of RAM. This is 1.5 times more than the de novo assembly with ALLPATHS-LG (231 GB) and 1.8 times more than the reference-guided de novo assembly with ALLPATHS-LG (195 GB). As IDBA-UD was originally developed to assemble small microbial genomes, the assembly algorithm is probably not memory optimized. This will strongly limit its application to smaller genomes. Lower memory requirements are a clear advantage, as not all labs have access to a large memory cluster. The reference-guided de novo assembly approach reduces the amount of required memory, due to the complexity reduction and break down of the de novo assembly step into many smaller ones. The reference-guided de novo with ALLPATHS-LG needs 16% less RAM than de novo assembly with ALLPATHS-LG. This is even more pronounced if the closer reference *A. lyrata* is used: only 109 GB memory is needed, which is less than half of the de novo assembly. However, the lower memory requirements of the reference-guided de novo assembly approach comes with the cost of run time, which is much longer due to several de novo assembly and alignment steps.

A further advantage of the reference-guided de novo assembly approach comes with the integration of de novo assemblies using multiple k-mers (Fig. 1, step 3: de novo assembly of superblocks and unaligned reads). De novo assemblers based on the de Bruijn graph often require the usage of a specific k-mer size (like ABySS or SOAPdenovo2), which is not that straightforward to choose [59]. Shorter k-mers leads to a loss of information and thus more ambiguities in the contig reconstruction. Additionally, repeats longer than the k-mer cannot be resolved. On the other hand, longer k-mers increase the risk that k-mers will not overlap or contain errors and thus break up contigs. Therefore, the combination of de novo assemblies using multiple k-mers can improve the reconstruction of genomes [59].

One of the main modifications we introduced into the reference-guided approach of Schneeberger et al. [19] is an additional de novo assembly step after the supercontig assembly (Fig. 1, step 5). This additional de novo assembly step makes it possible to rescue genome information of quite divergent regions, which in turn resolves the original within species limitation and allows the usage of a more distant and divergent genome to guide the assembly. In our simulation, we used *Arabidopsis thaliana* as a reference to guide the assembly of the *A. lyrata* genome. *A. thaliana* and *A. lyrata* are estimated to have diverged around ~5–22.7 million years ago and their genomes not only differ largely in size, but also in many rearrangements [35, 40, 41]. The evaluation in the low coverage simulation with and without the de novo assembly step 5 showed that our extension mostly improves the overall genome assembly and largely raises the completeness (see Fig. 6 and Additional file 2). Altogether, this demonstrates that with our approach even a related genome from a different species can be used to guide the de novo assembly and has the potential to improve the genome reconstruction. Of course, a less divergent genome leads to better results as can be seen in our simulations using *A. lyrata* as a reference (see refG2_ALLP in Fig. 2 and Additional file 1). It clearly outperformed all other assembly approaches. However, we used this as an extreme example since the reference and the assembled genome comes from the same species. In such cases, reads are often directly aligned against the reference genome and then an alternative consensus sequence is created. In any case, one should always use the closest available (and reliable) genome to guide the de novo assembly, since the closer the reference the better the results and the lower the memory requirements. Furthermore, the reference-guided de novo assembly may be improved by running it iteratively, in which the assembled genome is used as a reference in a next round of reference-guided de novo assembly [19] or in other reference guided algorithms like AlignGraph [20].

Besides all these, our study shows that longer assemblies or assemblies with a high N50 or NG50 are not always the best assemblies (see Fig. 3). Contigs or scaffolds maybe wrongly concatenated resulting in longer contigs/scaffolds and thus in artificially large N50 values [6, 56]. Comparing the ranking of NG50 and the GAGE corrected NG50 values already indicates large discrepancies. Especially, ALLPATHS-LG shows an extremely high NG50 value, which was probably caused by a lot of misjoined scaffolds. A conservative approach to solve this problem would be to split scaffolds with long Ns that lack synteny to a genome of a closely related species [60]. The comparison between Figs. 3, 4, and 5 illustrates that the ranking of the different assembly approaches can be quite different depending on the evaluation statistic in focus. Therefore, one should not judge an assembly based on a single metric, like the widely used N50, as an assembly may contain a lot of errors and misjoins [6, 7]. In our evaluation, we used a combination of 36 different statistics to analyze and rank the assembly approaches. These statistics integrate not only continuity and length measurements, but also assessments of accuracy and misjoins. However, most of these metrics can just be obtained if the genome sequence is known. In cases of de novo genome assemblies this is normally not the case and the evaluation gets much more difficult. Only a few tools try to detect assembly errors with the help of back mapped original reads (like ALE or REAPR) [55, 56] or infer the completeness with the presence of orthologous genes sets (like CEGMA or BUSCO) [52, 61]. We included some of these tools in our evaluation statistics. Anyhow, the evaluation without a true reference remains challenging [2] and often additional information from BAC/Fosmid sequences or optical maps are needed [2, 7].

In the future, long-read data will help to improve the assemblies by resolving large repetitive regions (which are also difficult to assemble with reference guided methods [22]), connect contigs into larger scaffolds and fill gaps of existing assemblies [7, 13, 62]. Unfortunately, their application is currently still limited by the high costs (especially for larger genomes) and error rates, but also by the more stringent DNA isolation requirements [1, 17]. However, this is expected to change in the near future.

## Conclusions

We have shown that our extended reference-guided de novo assembly approach almost always outperforms the corresponding de novo assembly program even when a reference genome of a closely related species is used. The combination of reference mapping and de novo assembly provides a powerful strategy for genome assembly, as it combines the advantages of both approaches [19, 20]. The reference-guided de novo assembly approach can be used with any de novo assembler, which allows the integration of the optimal de novo assembler for each species. Furthermore, an additional introduced de novo assembly step makes it possible to use a reference of a different species to guide the assembly. However, the reference genome should be as close as possible, as better results can be obtained and the memory requirements are reduced. Overall, the evaluation of an assembly is not straightforward and single measurements (like the N50) can be misleading. Therefore, one should always use an elaborated combination of evaluation metrics to compare different assembly programs and approaches.

## Additional files

Additional file 1: Table of evaluation statistics. (XLSX 47 kb)

Additional file 2: Table of evaluation statistics at low coverage. (XLSX 51 kb)
